# Cross-Contamination of a UROtsa Stock with T24 Cells – Molecular Comparison of Different Cell Lines and Stocks

**DOI:** 10.1371/journal.pone.0064139

**Published:** 2013-05-17

**Authors:** Georg Johnen, Peter Rozynek, Yvonne von der Gathen, Oleksandr Bryk, Ricarda Zdrenka, Christian Johannes, Daniel G. Weber, O′Brien Igwilo-Okuefuna, Irina Raiko, Jörg Hippler, Thomas Brüning, Elke Dopp

**Affiliations:** 1 Institute for Prevention and Occupational Medicine of the German Social Accident Insurance, Institute of the Ruhr University Bochum (IPA), Bochum, Germany; 2 Institute of Hygiene and Occupational Medicine, University Hospital Essen, Essen, Germany; 3 Department of Genetics, University of Duisburg-Essen, Essen, Germany; 4 Institute of Environmental Analytical Chemistry, University of Duisburg-Essen, Essen, Germany; 5 Zentrum für Wasser- und Umweltforschung, University of Duisburg-Essen, Essen, Germany; St. Georges University of London, United Kingdom

## Abstract

**Background:**

UROtsa is an authentic, immortalized human urothelial cell line that is used to study the effects of metals and other toxic substances, mostly in the context of bladder cancer carcinogenesis. Unusual properties on the molecular level of a provided UROtsa cell line stock prompted us to verify its identity.

**Methods:**

UROtsa cell line stocks from different sources were tested on several molecular levels and compared with other cell lines. MicroRNA and mRNA expression was determined by Real-Time PCR. Chromosome numbers were checked and PCR of different regions of the large T-antigen was performed. DNA methylation of *RARB, PGR, RASSF1, CDH1, FHIT, ESR1, C1QTNF6*, *PTGS2, SOCS3*, *MGMT*, and *LINE1* was analyzed by pyrosequencing and compared with results from the cell lines RT4, T24, HeLa, BEAS-2B, and HepG2. Finally, short tandem repeat (STR) profiling was applied.

**Results:**

All tested UROtsa cell line stocks lacked large T-antigen. STR analysis unequivocally identified our main UROtsa stock as the bladder cancer cell line T24, which was different from two authentic UROtsa stocks that served as controls. Analysis of DNA methylation patterns and RNA expression confirmed their differences. Methylation pattern and mRNA expression of the contaminating T24 cell line showed moderate changes even after long-term culture of up to 56 weeks, whereas miRNAs and chromosome numbers varied markedly.

**Conclusions:**

It is important to check the identity of cell lines, especially those that are not distributed by major cell banks. However, for some cell lines STR profiles are not available. Therefore, new cell lines should either be submitted to cell banks or at least their STR profile determined and published as part of their initial characterization. Our results should help to improve the identification of UROtsa and other cells on different molecular levels and provide information on the use of urothelial cells for long-term experiments.

## Introduction

UROtsa cells are a valuable tool to study toxic effects and the development of urothelial cancers. Especially the carcinogenic effects of arsenic have been studied using the UROtsa cell model [1]. Arsenic is considered to be the most harmful toxin in drinking water worldwide and therefore constitutes a major public health problem [2]. The UROtsa cell line was generated by immortalization of urothelial cells with a construct containing the SV40 large T-antigen [3]. It is an authentic and well-characterized cell line [3]–[5]. In contrast to cells immortalized with live SV40 virus (SV-HUC-1, SV-HUC-2) [6] UROtsa cells have the advantage of a stable karyotype and show no indication of anchorage-independent growth in later passages [3]. The cells also do not form tumors in immunocompromised mice. In that regard, UROtsa is unique among the urothelial cell lines. Despite of being derived from the urothelial lining of the ureter UROtsa is considered to be a useful model for normal human bladder urothelium [1], [3], [4]. The urothelium (transitional epithelium) consists of stratified cell layers that line the urinary passages, i.e., the renal pelvis, the ureters, the urinary bladder, and the proximal urethra [7]. The urothelia of the different anatomical sites share a similar morphology but have different developmental origins and consequently are distinct in a number of biochemical and ultrastructural features [8]. The urothelium can be divided into at least three different lineages (renal pelvis/ureter, bladder, and proximal urethra), with the urothelium of the renal pelvis/ureter/trigone deriving from the mesoderm and the bladder/urethra from the endoderm [9]. In contrast to cells from the ureter, creating immortalized cell lines from bladder urothelium is more difficult [8]. This may explain the paucity of immortalized non-malignant cell lines from bladder urothelium.

The UROtsa cell line is easy to maintain, proliferates in serum-containing medium, and requires no feeder cells. It is relatively undifferentiated and only forms a monolayer instead of the stratified layers that primary cells are able to form. Whereas in serum-free medium, UROtsa cells have been induced to partially differentiate to structures resembling the intermediate layer of bladder urothelium [4]. Unfortunately, there is always the trade-off between proliferation and high differentiation. So far, a human uroepithelial cell line that features a fully differentiated, stratified bladder epithelium as well as the potential of unlimited serial growth has not been described. Primary cultures are highly differentiated but have only very limited growth potential. Unlimited growth potential is necessary to mimic chronic exposure to carcinogens in long-term experiments that last, for example, up to one year [1], [10].

As a non-malignant cell line with the possibility to perform long-term studies, UROtsa represents a good compromise. UROtsa can therefore be used to study mechanisms of carcinogenesis, including early steps of malignant transformation and the search for biomarkers for the early detection of bladder cancer [1], [11], [12]. In contrast, (bladder) cancer cell lines are not suitable to study early molecular changes during carcinogenesis [13]. These cell lines would be better suited to study cancer progression, metastasis etc., or the evaluation of chemotherapeutic agents. A number of human bladder cancer cell lines are available from cell banks in Australia, Germany, Japan, and the USA: 5637, 647-V, BC-3C, BFTC-905, CAL-29, HT-1197, HT-1376, J82, JMSU-1, KMBC-2, KU-19-19, RT112, RT4, SCaBER, SW-1710, SW-780, T24, TCC-SUP, U-BLC1, UM-UC-1, −3, −5, −6, −7, −9, −10, −11, −16, and VM-CUB1. Cell lines derived from carcinomas of the renal pelvis are KMPC-3 and UM-UC-14. Available ureter-derived cell lines are 639-V, Hs 789.T, MC-SV-HUC T-2, and SV-HUC-1 (www.cellbankaustralia.com, www.dmsz.de, cellbank.nibio.go.jp, www.atcc.org). UROtsa or other immortalized and non-malignant urothelial cell lines are, to our knowledge, not available from the large repositories. However, other immortalized urothelial cell lines, like TERT-NHUC and TRT-HU1, have been described in the literature [14], [15].

Cross-contamination of cell lines has a long history [16]. The most frequent contaminations are attributed to HeLa cells, followed by T24 [17]. The introduction of short tandem repeat (STR) profiling has greatly improved proper identification of cell lines [18], [19]. However, regular verification of cell lines is still not a standard procedure, in part because the required methodologies are not always easily accessible or simply because of unawareness of the problem. Therefore, many “false” cell lines continue to be persistently used, leading to tainted publications and a waste of time and public money [16], [19], [20].

Originally, we intended to use UROtsa cells to study the effects of long-term exposure to arsenic compounds and to induce neoplastic transformation, similar to the experiments of Sens et al. and Jensen et al. [10], [21]. While studying the effects of long-term tissue culture on unexposed UROtsa cells that served as a control, we noticed an unusual expression of several genes and a high degree of DNA methylation of several tumor suppressor genes. Further investigation led to the discovery that the supposed UROtsa cells were in fact a different cell line. Here, we present evidence for the cross-contamination of a widely distributed UROtsa cell line stock by T24 cells.

## Materials and Methods

### Cells and cell culture reagents

Table 1 lists all cell lines and stocks used in the study. Cell culture medium for UROtsa consisted of Earle's minimal essential medium (EMEM) (c.c.pro GmbH, Oberdorla, Germany) enriched with 10% fetal bovine serum (FBS) (GIBCO, Darmstadt, Germany), 0.5% gentamycin (c.c.pro) and 1% L-glutamine (c.c.pro). Growth medium for HepG2 additionally contained 1% not essential amino acids and 1% sodium pyruvate (c.c.pro). T24 cells were grown in DMEM/Ham's F12 (1∶1) (c.c.pro) with 0.5% gentamycin, 1% L-glutamine, 15 mM Hepes, and 5% FBS. HeLa S3 were grown in Ham's F12 with 2 mM L-glutamine and 10% FBS. RT4 were grown in McCoy's 5a with 10% FBS. BEAS-2B were grown in RPMI 1640 (c.c.pro) with 10% FBS. All cells were maintained in a humidified incubator with 5% CO_2_ at 37°C. For detachment of cells 0.25% Trypsin-EDTA (c.c.pro) was used.

**Table 1:** Cell lines used in the study.

Name of cell line or cell line stock	Number of cell line	Primary tissue	Real identity	Source (Institution)	Address	Year of acquisition	Origin and history**
UROtsa-1	–	Immortalized normal urothelium	UROtsa	J.R. Masters (University College London)	London, UK	2011	[3]
UROtsa-2	–	Immortalized normal urothelium	(UROtsa)***	S.H. Garrett (University of North Dakota)	Grand Forks, ND	2005	[3], [4]
UROtsa-3	–	Transitional cell carcinoma of the urinary bladder**	T24	M. Styblo (University of North Carolina)	Chapel Hill, NC	2008	[3], [76], [77]
UROtsa-4	–	Immortalized normal urothelium	UROtsa	A. Fabarius (University of Heidelberg)	Mannheim, Germany	2011	[3], [5], [78]
UROtsa/F35	–	Transduced UROtsa-3**	T24	M. Styblo (University of North Carolina)	Chapel Hill, NC	2011	[3], [25], [76], [77]
T24	ACC 376	Transitional cell carcinoma of the urinary bladder	–	DSMZ	Braunschweig, Germany	2011	–
HeLa S3	ACC 161	Cervix carcinoma	–	DSMZ	Braunschweig, Germany	2011	–
HepG2	HB-8065	Hepatocellular carcinoma	–	ATCC	Manassas, VA	2003	–
RT4	HTB-2	Well-differentiated transitional papillary tumor of the urinary bladder	–	ATCC	Manassas, VA	2006	–
BEAS-2B	CRL-9609	Immortalized normal bronchial epithelium	–	ATCC	Manassas, VA	2011	–
HUEPC	536×090206* 459×250403*	Primary urothelium	–	Provitro GmbH	Berlin, Germany	2012	–

DSMZ: German Collection of Microorganisms and Cell Cultures, ATCC: American Type Culture Collection.

* Lot numbers.

** All UROtsa stocks originated in the lab of I.M. Leigh and J.R. Masters [3] and were then handed from lab to lab, as described in the cited literature. We created the numbering to distinguish the different stocks. UROtsa-3 was cross-contaminated with T24 at some time point and UROtsa/F35 was derived from UROtsa-3. Therefore, both are not authentic UROtsa cell line stocks but T24 cells. Based on STR profiling only UROtsa-1 and UROtsa-4 are authentic.

*** UROtsa-2 was not tested by STR profiling but showed (in contrast to T24) to have normal chromosomal numbers like authentic UROtsa.

The primary urothelial cells HUEPC (Provitro, Berlin, Germany) were directly used for analysis and served as a control. Exfoliated cells obtained from normal human urine served as another control. The study was approved by the ethics committee of the Ruhr University Bochum (No. 3674-10). All participants gave written informed consent. Urine (40 ml) was collected from four healthy donors. Cells from urine were harvested by centrifugation at 500× g for 10 min at 10°C [22].

Stocks of the original UROtsa cell line (thereafter called “UROtsa-1”) have been known to be contaminated by Mycoplasma at least since 2001 (J. R. Masters, personal communication; M. Styblo, personal communication). Unfortunately, another stock (“UROtsa-2”) showed also indications of an infection when we received it in 2005. We therefore used a stock (“UROtsa-3”) from a source where the cells had been treated and cured of the Mycoplasma infection (M. Styblo and Z. Drobna, personal communication). Of UROtsa-3 we received two vials in 2008. One was taken into culture in 2008 and was used for long-term experiments (sample A), the other was first cultured in 2011 and served as a control (sample B). After a cross-contamination of UROtsa-3 with an unknown cell line became more likely, another UROtsa stock (“UROtsa-4”) was acquired, which was proven to be free of Mycoplasma and possible cross-contaminations [5]. To reduce the risk of Mycoplasma infection in the lab, UROtsa-1 was only used for STR analysis and UROtsa-2 for chromosomal analysis.

Cells from the UROtsa-3 stock were maintained in long-term culture for 56 weeks and UROtsa-4 cells for 34 weeks. Samples were harvested every four weeks (UROtsa-4: every two weeks) to study molecular parameters. Harvested cells were either kept in PBS (for analysis of genomic DNA and CpG methylation) or RNAlater (for RNA analysis) at −20°C until analysis. Unless otherwise stated, experiments were done with cells derived from sample A of UROtsa-3.

### Detection of Mycoplasma

Mycoplasma detection was performed with a commercial kit that is based on a quantitative PCR method that covers a broad range of Mycoplasma species. DNA (30 ng per sample) was isolated from cells (see below) and amplified with the PromoKine PCR Mycoplasma Test Kit I/RT from PromoCell GmbH (Heidelberg, Germany). Reactions were run in duplicate with Variant A of the kit on a BioRad CFX96 (Bio-Rad Laboratories GmbH, Munich, Germany) according to the manufacturer's instructions. The detection limit is 10–15 fg of Mycoplasma DNA. All cell line stocks were tested. Only the UROtsa-2 stock showed a positive result. In addition, during the long-term experiments, cells sampled at the following time points were tested: week 4, 28, and 56 (UROtsa-3) and week 2, 14, and 34 (UROtsa-4). All time points showed a negative result.

### RNA extraction

Cells were stored in RNAlater (Ambion, Austin, TX) immediately after harvest. Total RNA was isolated from 10^6^ cells per sample using the mirVana miRNA Isolation Kit (Ambion) according to the manufacturer's instruction. Concentration of total RNA was measured using a NanoDrop ND-1000 spectrophotometer (PEQLAB, Erlangen, Germany). RNA quality was determined on a 2100 Bioanalyzer using microfluidic “RNA Nano 6000 Chips” (Agilent Technologies, Waldbronn, Germany).

### Analysis of mRNA expression

Individual TaqMan mRNA Assays (Applied Biosystems, Darmstadt, Germany) were used to analyze the expression of the following mRNAs: *ZEB1* (Hs00232783_m1), *CDH1* (Hs01023895_m1), *KRT17* (Hs01588578_m1), *KRT20* (Hs00300643_m1), *UPK1A* (Hs01086736_m1), *VIM* (Hs00185584_m1), *TP53* (Hs01034249_m1), *RB1* (Hs01078066_m1), *TP63* (Hs00979340_m1), *HRAS* (Hs00610483_m1), *NOTCH1* (Hs01062014_m1), and *GAPDH* (Hs99999905_m1). Quantitative Real-Time PCR was performed using a 7900 HT Fast Real-Time PCR System (Applied Biosystems) according to the manufacturer's instructions. 10 ng total RNA and 5 µl cDNA were used as templates for the RT and PCR reaction, respectively. Samples were analyzed in duplicate and non-template controls were included in all assays.

The ABI TaqMan SDS v2.3 software was used to obtain raw Ct values. For all samples the baseline was adjusted automatically and the threshold set manually to 0.2. All mRNAs with Ct values greater than 35 were set to 35 and considered non-detectable. Relative quantification of mRNA expression was calculated using RQ manager v1.2. *GAPDH* mRNA served as endogenous control for normalization. Relative expression levels were expressed as 2^−dCt^, with dCt  =  Ct_(*X*)_-Ct_(*GAPDH*)_. Raw data can be found in supplemental Table S1.

### Analysis of microRNA (miRNA) expression

Individual TaqMan miRNA Assays (Applied Biosystems) were used to analyze the expression of the following miRNAs: miR-141 (000463), miR-200a (000502), miR-200b (002251), miR-200c (000505), and miR-429 (001024) on a 7900 HT Fast Real-Time PCR System according to the manufacturer's instructions. 10 ng total RNA and 5 µl cDNA were used as templates for the RT and PCR reaction, respectively. Samples were analyzed in duplicate and non-template controls were included in all assays. The ABI TaqMan SDS v2.3 software was used to obtain raw Ct values. For all samples the baseline was adjusted automatically and the threshold set manually to 0.2. All miRNAs with Ct values greater than 35 were set to 35 and considered non-detectable. The RQ manager v1.2 was used for relative quantification of miRNA expression. The mean of RNU44 (001094) and RNU48 (001006) served as endogenous control for normalization. Relative expression levels are shown as 2^−dCt^. Raw data can be found in supplemental Table S1.

### Analysis of large T-antigen

To detect fragments of SV40 large T-antigen in the genome of UROtsa cells we used a PCR-based method. We designed three primer pairs spanning different regions of the large T gene: 5′-region (bp 5136 - 4958 in SV40, Acc.-No. J02400) primers LT5-5: 5′-TCTTTGCAGCTAATGGACCTTCTA, LT5-3: 5′-GCATATTTTACTCCATCTTCCATT, middle region (bp 4483 - 4052) primers LTM-5: 5′-GTGATGATGAGGCTACTGC, LTM-3: 5′-CATGCTCCTTTAACCCACCTG, and 3′-region (bp 3144 - 2702) primers LT3-5: 5′-ACGCAGTGAGTTTTTGTTAGA, LT3-3: 5′-GTTCAGGGGGAGGTGTGG. The PCR reaction (20 µl) contained 2.5 mM MgCl_2_, 10 pmol of each primer, 200 µM of each dNTP, and 1.5 U AmpliTaq Gold in PCR Gold Buffer (Life Technologies, Darmstadt, Germany). PCR conditions were as follows: 9 min at 95°C, 33 cycles of 30 sec 95°C/30 sec 60°C/30 sec 72°C, and a final step of 5 min at 72°C/hold at 8°C. PCR products were visualized on a 2% agarose gel (Agarose NEEO; Roth, Karlsruhe, Germany) in 1× TBE-buffer (Rotiphorese; Roth) stained with Roti®-Safe GelStain (Roth; 5 µl/100 ml agarose solution).

To detect expression of large T-antigen mRNA was isolated and reverse transcribed. The resulting cDNA was analyzed by PCR with primer pairs (F: 5′-AATAGCAAAGCAAGCAAGAGT-3′, R: 5′-GAAAATGGAAGATGGAGTAAA-3′ and F: 5′-TTCATGCCCTGAGTCTTCCAT-3′, R: 5′-GCCAGGAAAATGCTGATAAAAATG-3′) and conditions described by Dube et al. and Stone et al., respectively [23], [24]. PCR products were visualized on a Bioanalyzer with a microfluidic DNA 1000 chip (Agilent).

### DNA isolation and CpG methylation analysis

DNA was isolated from 2×10^6^ cells with the QIAamp® DNA Mini Kit (Qiagen, Hilden, Germany). The cells were centrifuged at room temperature for 5 min at 500× g and the pellet was resuspended in 180 µl ATL-Buffer; 20 µl proteinase K was added and incubated at 56°C for 60 min. The following steps were performed using a QIAcube automated workstation (Qiagen) with the protocol “Purification of DNA from tissues” (elution volume 200 µl). Concentration of DNA was measured using a NanoDrop ND-1000 and visualized on a 1% agarose gel.

Bisulfite conversion was performed using the EpiTect Bisulfite Kit (Qiagen) according to the instructions of the manufacturer with an extension of the bisulfite conversion thermal-cycling conditions by adding a denaturation step of 5 min at 95°C followed by 2 h at 60°C before the final step (hold 20°C). The cleanup was performed using the QIAcube (Qiagen) with the protocol “Cleanup of bisulfite converted DNA”.

Gene promoter regions of *LINE1, RARB, PGR (PGRB), RASSF1, CDH1, FHIT, ESR1, C1QTNF6, PTGS2, SOCS3, and MGMT* were amplified using the PCR conditions and primer sequences specified in the supplemental information (Table S2). Each PCR product was visualized on a 2% agarose gel to check the correct size of the amplicon. The methylation status of the regions of interest was analyzed by pyrosequencing using the PyroMark Q96 Vacuum Prep Workstation (Qiagen) and the PyroMark Q96 ID pyrosequencer with PyroMark Gold Q96 reagents (Qiagen). The analysis was performed at least twice for each promoter region.

### Analysis of chromosomal numbers

For analysis of numerical chromosomal aberrations UROtsa cells were harvested from subconfluent cultures after addition of 0.08 µg/ml colcemid (Ciba, Basel, Switzerland) for the last 2 h. Metaphase spreads were stained with Giemsa (5% solution in phosphate buffer). The chromosome numbers were counted in 30 metaphases per passage or cell line stock.

### STR profiling

To identify the different cell lines and stocks the PowerPlex 16 Kit (Promega, Madison, WI) was used for STR profiling. The STR profiling was performed according to the manufacturer's instructions using the protocols for the GeneAmp® PCR System 2400 Thermal Cycler (Perkin Elmer/Applied Biosystems, Waltham, MA) and the ABI PRISM® 310 Genetic Analyzer (Perkin Elmer/Applied Biosystems).

STR profiles were analyzed with the software package Genemapper 4.1 (Applied Biosystems, Carlsbad, CA). The STR profile of UROtsa cell had been determined before but was never published in detail [5]. A. Fabarius kindly provided us with this previous analysis of UROtsa-4 cells that had been performed at the DSMZ in 2009. Results were compared with official entries of different cell lines in the STR profile database maintained by DSMZ (German Collection of Microorganisms and Cell Cultures, www.dsmz.de).

## Results

### Expression of mRNAs and miRNAs as well as levels of DNA methylation during long-term culture of UROtsa stocks

To study the time course of several molecular parameters we maintained a long-term culture of unexposed UROtsa-3 cells for 56 weeks. Parameters were determined in intervals of four weeks. On the mRNA level, the expression of *TP53*, *RB1*, *HRAS*, and *NOTCH1* showed little changes over time, while *VIM* showed an initial increase and *ZEB1* a continuous but moderate decrease; *KRT17* varied on a low level (Figure S1). We could not detect mRNA expression of the uroepithelial markers *CDH1*, *KRT20*, or *TP63* at any time point of the long-term experiment, while *UPK1A* was detectable (on low level) only at a few time points (Table S1).

**Figure 1:**
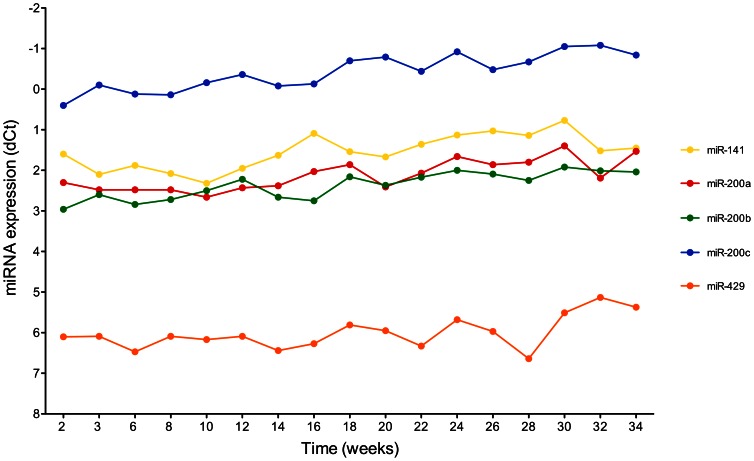
Time course of miRNA expression (miR-200 family) during long-term culturing of authentic UROtsa-4 cells. Normalized levels of miR-141 (yellow), miR-200a (red), miR-200b (green), miR-200c (blue), and miR-429 (orange) are shown as determined by Real-Time PCR. RNU44 and RNU48 were used for normalization.

The miR-200 family, comprising miR-200a, miR-200b, miR-200c, and miR-429, showed similar expression profiles of its individual members (miR-141 was not detectable). Starting with the twelfth week, the expression increased until the 20th/24th week and largely remained on a plateau in UROtsa-3 cells until the 56th week (Figure S2). For comparison, miRNAs were also determined during long-term culture (34 weeks) of UROtsa-4 cells. Here, all five members of the miR-200 family showed only a minor increase in expression and had, in general, higher expression levels (Figure 1).

A major goal of the original long-term experiment was to study changes in the degree of DNA methylation of CpG islands in the promoter region of several cancer-related genes in UROtsa cells. Figure S3 depicts the time course of the methylation of 11 different genes as determined by pyrosequencing in UROtsa-3. The methylation levels of *LINE1, RARB, PGR, RASSF1, CDH1, C1QTNF6*, *PTGS2, SOCS3*, and *MGMT* remained basically stable during a time period of up to 56 weeks. The promoter methylation of *FHIT* and *ESR1* showed an increase from about 34% to 53% and 32% to 45%, respectively.

### Profiles of mRNA and miRNA expression as well as of DNA methylation in different cell lines and UROtsa stocks

**Figure 2:**
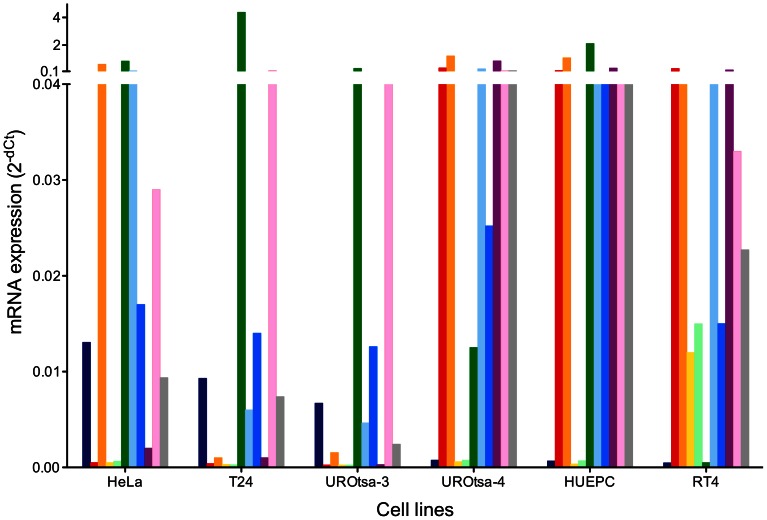
Comparison of mRNA expression in different cell lines and UROtsa stocks. Normalized mRNA levels of *ZEB1* (dark blue), *CDH1* (red), *KRT17* (orange), *KRT20* (yellow), *UPK1A* (light green), *VIM* (green), *TP53* (light blue), *RB1* (blue), *TP63* (purple), *HRAS* (pink), and *NOTCH1* (grey) were determined in HeLa, T24, UROtsa-3/T24, UROtsa-4, primary urothelial cells (HUEPC), and RT4. Expression levels were determined by Real-Time PCR. *GAPDH* mRNA was used for normalization. Ct values >35 were considered as not detectable, usually resulting in 2^−dCT^ values below 0.001. The y axis was adapted to better accommodate the genes with low expression levels. Original data can be found in Table S1.

To confirm the lack of uroepithelial markers and to compare the expression of genes the mRNA determination of *ZEB1*, *CDH1, KRT17, KRT20, UPK1A, VIM, TP53, RB1, TP63*, *HRAS*, and *NOTCH1* was repeated for UROtsa-3 cells and compared with other UROtsa stocks and other cell lines. Figure 2 shows the mRNA expression levels of UROtsa-3, UROtsa-4, the cervical carcinoma cell line HeLa, the urothelial carcinoma cell line T24, the papillary tumor cell line RT4, and a primary urothelial cell line (HUEPC) as determined by Real-Time PCR. Each cell line had a unique expression pattern. UROtsa-3 showed an mRNA profile that was markedly different from UROtsa-4 and HUEPC but resembled T24. *KRT20* and *UPK1A* could only be detected in the well-differentiated papillary tumor cell line RT4.

**Figure 3:**
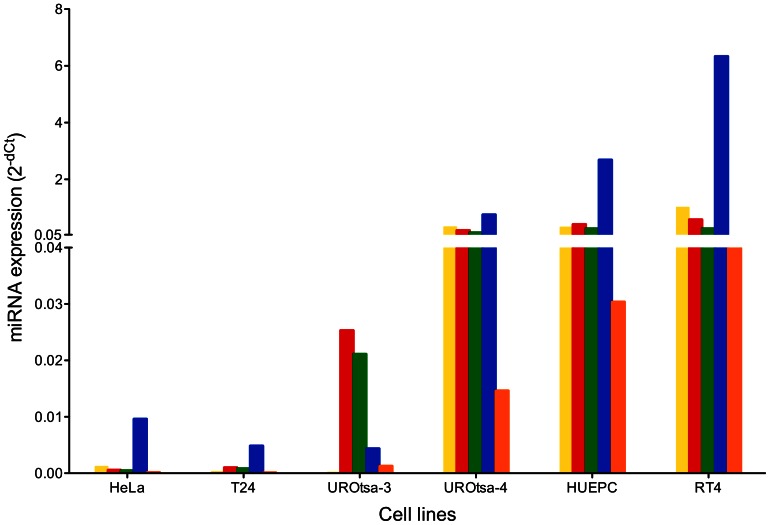
Comparison of miRNA expression in different cell lines and UROtsa stocks. Normalized levels of miR-141 (yellow), miR-200a (red), miR-200b (green), miR-200c (blue), and miR-429 (orange) were determined in HeLa, T24, UROtsa-3/T24, UROtsa-4, primary urothelial cells (HUEPC), and RT4. Expression levels were determined by Real-Time PCR. RNU44 and RNU48 were used for normalization. Ct values >35 were considered as not detectable, usually resulting in 2^−dCT^ values below 0.0002. The y axis was adapted to better accommodate the genes with low expression levels.

The miR-200 family showed an expression profile for each cell line with similarities between UROtsa-4, HUEPC, and RT4 while in UROtsa-3, HeLa, and T24 the miRNAs were barely detectable (Figure 3).

**Figure 4:**
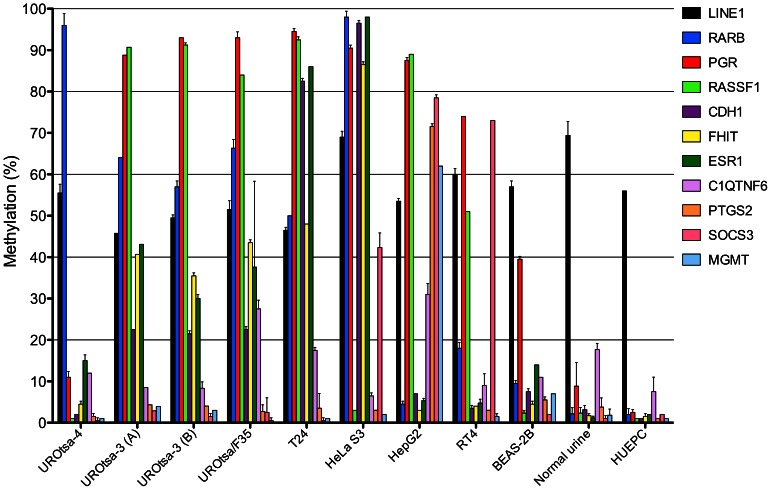
Comparison of DNA methylation patterns in different cell lines and UROtsa stocks. The degree of promoter methylation of the genes *LINE1, RARB, PGR, RASSF1, CDH1, FHIT, ESR1, C1QTNF6*, *PTGS2, SOCS3*, and *MGMT* was determined by pyrosequencing in the cell lines and stocks UROtsa-4, UROtsa-3/T24 (sample A and B), UROtsa/F35, T24, HeLa, HepG2, RT4, BEAS-2B, as well as sedimented cells from normal urine and primary urothelial cells (HUEPC). Note: BEAS-2B cells have been cultured in 10% FBS that causes squamous differentiation and might therefore influence methylation patterns [45].

To determine whether DNA methylation analysis could distinguish different cell lines and identify possible cross-contaminants we investigated a panel consisting of *LINE1, RARB, PGR, RASSF1, CDH1, FHIT, ESR1, C1QTNF6*, *PTGS2, SOCS3*, and *MGMT* with UROtsa and other cell lines. Figure 4 shows the methylation pattern of UROtsa-3 (two independent samples), UROtsa/F35 (a clonal variant of UROtsa-3), and UROtsa-4, the cell lines HeLa, HepG2, BEAS-2B, RT4, and T24 as well as a primary urothelial cell line (HUEPC) and normal human urothelial cells from the urine of four healthy donors. In the investigated cells the methylation of the retroposon *LINE1* ranged between 46% and 69% and was somewhat decreased in comparison to the normal primary cells (Table 2). In contrast, methylation of the other ten genes, which had a low level of methylation in normal urothelial cells, was markedly increased in some cell lines. Each cell line or stock had a unique methylation pattern and UROtsa-3 clearly showed a different methylation pattern than UROtsa-4. Only the two samples of UROtsa-3 and its variant UROtsa/F35 had a very similar pattern. In general, UROtsa-3 exhibited a relatively high degree of DNA methylation, similar to the cancer cell lines HeLa and HepG2 but mostly resembling T24. The less aggressive RT4 cell line and even more the immortalized non-malignant cell lines BEAS-2B and UROtsa-4 had a lower degree of CpG methylation. Primary urothelial cells showed the lowest CpG methylation in the targeted promoter regions.

**Table 2:** DNA methylation in different cell lines and UROtsa stocks.

	Gene promoter region (% methylation)										
Cell line	LINE1	RARB	PGR	RASSF1	CDH1	FHIT	ERS1	C1QTNF6	PTGS2	SOCS3	MGMT
UROtsa- 4	56	96	11	1	2	5	15	12	2	1	1
UROtsa-3 (A)	46	64	89	91	23	41	42	9	2	3	4
UROtsa-3 (B)	50	57	93	91	22	36	30	8	4	2	3
UROtsa/F35	52	66	93	84	23	44	38	28	3	3	1
T24 (ACC 376)	47	50	95	93	83	48	86	18	2	1	1
HeLa S3 (CCL-2.2)	69	98	91	3	97	87	98	7	3	43	2
HepG2 (HB-8065)	54	5	88	89	7	3	5	31	72	79	62
RT4 (ACC 412)	60	18	74	51	4	4	5	9	3	73	2
BEAS-2B (CRL-9609)	57	10	40	2	8	5	14	11	6	2	7
Urothel urine*	69	2	9	2	3	2	1	18	4	1	2
HUEPC	56	2	3	1	1	2	2	8	1	2	1

* Mean of results obtained from four different healthy persons.

### Lack of SV40 large T-antigen sequences in original UROtsa cells

**Figure 5:**
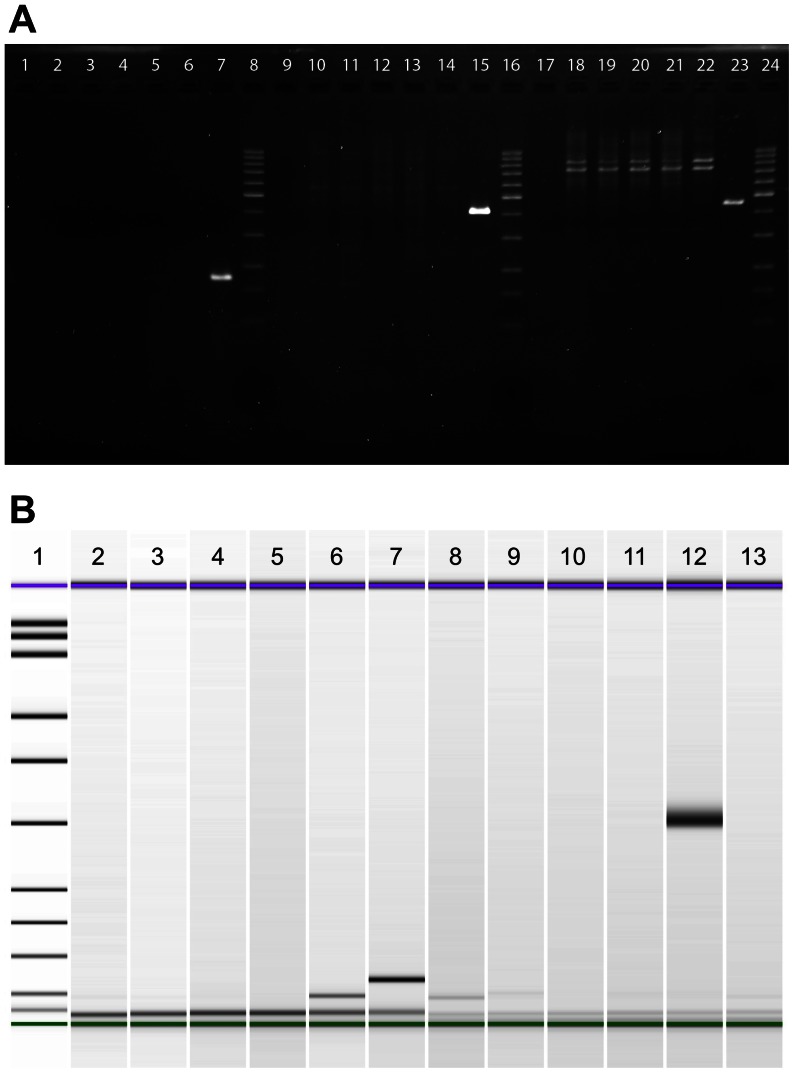
PCR analysis of cell lines and UROtsa stocks to detect large T-antigen. (A) PCR reactions were resolved on a 2% agarose gel. *SV40gp6* sequences (5′-, 3′-, and middle part) are absent in UROtsa-1 (lanes 2, 10, 18), UROtsa-3/T24 (lanes 3, 11, 19), UROtsa-4 (lanes 4, 12, 20), HeLa (negative control, lanes 5, 13, 21) and T24 (negative control, lanes 6, 14, 22) but not in BEAS-2B (positive control, lanes 7, 15, 23). No-template controls are in lanes 1, 9, and 17. A 100 bp ladder served as size marker (lanes 8, 16, 24). (B) PCR reactions of reverse-transcribed mRNA were resolved on a microfluidic DNA 1000 chip (Bioanalyzer). Large T-antigen expression (expected product size: 65 bp in lanes 2–7 [24] and 304 bp in lanes 8–13 [23]) is absent in UROtsa-3/T24 (lanes 3 and 8), UROtsa-4 (lanes 4 and 9), HeLa (negative control, lanes 5 and 10), and T24 (negative control, lanes 6 and 11) but not in BEAS-2B (positive control, lanes 7 and 12). A no-template control was loaded in lanes 2 and 13. Size markers (lane 1) are 15 (green), 25, 50, 100, 150, 200, 300, 400, 500, 700, 850, 1000, and 1500 bp (purple).

A quick experiment to check for the identity of UROtsa would have been to detect the SV40 large T-antigen gene (*SV40gp6*) in the genomic DNA of the cells. Interestingly, none of the UROtsa cell line stocks showed any sequences of *SV40gp6*, while the SV40-transformed lung cell line BEAS-2B showed a clear signal in all PCR assays (Fig. 5A). We used three different primer pairs, covering the 5-’, 3′-, and middle part of the gene. The lack of large T-antigen in UROtsa was also demonstrated on the expression level with two primer pairs specific for mRNA after reverse transcription (Fig. 5B).

### Chromosomal numbers in UROtsa cell line stocks

**Figure 6:**
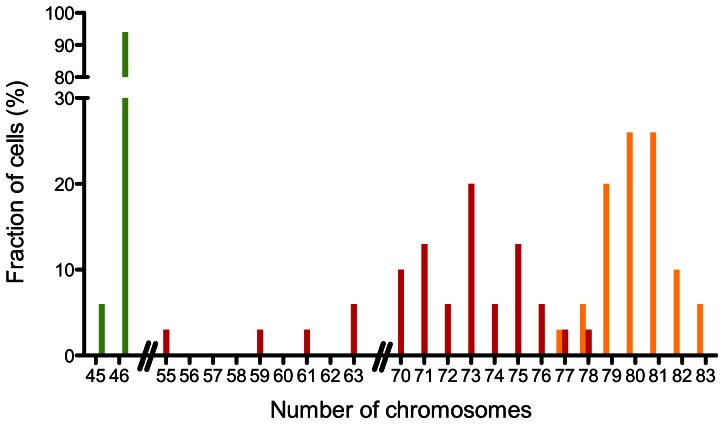
Chromosome numbers of UROtsa cell line stocks. In contrast to the normal (46, XX) karyotype of UROtsa cells [3], [5] the UROtsa-3/T24 stock had a median of 80 chromosomes (early passage). Shown is the distribution of chromosomes for an early (P5, week 3; orange) and a later passage (P39, week 32; red). The normal chromosome numbers of authentic UROtsa could be confirmed for the UROtsa-2 (not shown) and UROtsa-4 stock (green).

Most cancer cell lines show aneuploidy. To check whether UROtsa-3 cells show indications of a neoplastic transformation on the cytogenetic level we looked for possible numerical aberrations of their chromosomes in metaphase spreads. The normal karyotype of UROtsa-4 has been confirmed recently [5]. Accordingly, UROtsa-2 and UROtsa-4 cells had normal appearing metaphases of 46 chromosomes. In contrast, the chromosome numbers of an early passage (P5, week 3) of UROtsa-3 indicated aneuploidy with a median of 80 chromosomes. A later passage (P39, week 32) showed a broader distribution of chromosome numbers with a median of 73, indicating major changes during culturing (Fig. 6).

### STR analysis reveals cross-contamination

To finally determine the identity of the UROtsa-3 cell line stock we applied STR profiling. We tested UROtsa-1, UROtsa-3, UROtsa/F35, UROtsa-4, and for comparison, T24 cells, which were the most likely contaminant of our cell line stock. We employed a kit that allowed the analysis of 16 different STRs. Our analysis confirmed that UROtsa-1 and UROtsa-4 were basically identical, with a small difference at one of the alleles in *D21S11* and *Penta* D (Table 3; Supplemental Figure S4). As expected, our analysis of UROtsa-4 was 100% identical to the unpublished DSMZ analysis (A. Fabarius, personal communication). In contrast, UROtsa-3 was markedly different from UROtsa-1 and UROtsa-4. Instead, the STR profile of both UROtsa-3 samples was 100% identical to a DSMZ database entry of T24 (HTB-4) and very similar (difference in one allele of *vWA*) to the T24 cell line (ACC 376) used for comparison. Interestingly, our T24 (ACC 376) and the corresponding database entry for ACC 376 showed a difference in one of the D13S317 alleles. UROtsa/F35, a clonal variant that was created from UROtsa-3 to express the gene *ASMT3* [25], [26], also had an STR profile identical to UROtsa-3 and T24. UROtsa/F35, which we received in 2011, served as an additional control to exclude a cross-contamination in our lab.

**Table 3:** STR profiling of different UROtsa cell line stocks and T24 cells.

	STR locus															
Cell line	Amelogenin	CSF1PO	D13S317	D16S539	D5S818	D7S820	TH01	TPOX	vWA	Penta E	D18S51	D21S11	D3S1358	FGA	D8S1179	Penta D
UROtsa- 1	XX	11, 12	11, 13	11, 12	12, 13	11, 12	6, 9.3	8, 9	16, 16	11, 17	16, 16	27, 29	15, 15	20, 20	14, 14	11, 12
UROtsa- 4	XX	11, 12	11, 13	11, 12	12, 13	11, 12	6, 9.3	8, 9	16, 16	11, 17	16, 16	29, 29	15, 15	20, 20	14, 14	11, 11
UROtsa-3 (A)	XX	10, 12	12, 12	9, 9	10, 12	10, 11	6, 6	8, 11	17, 17	7, 10	16, 18	29, 29	16, 16	17, 22	14, 14	11, 15
UROtsa-3 (B)	XX	10, 12	12, 12	9, 9	10, 12	10, 11	6, 6	8, 11	17, 17	7, 10	16, 18	29, 29	16, 16	17, 22	14, 14	11, 15
UROtsa/F35	XX	10, 12	12, 12	9, 9	10, 12	10, 11	6, 6	8, 11	17, 17	7, 10	16, 18	29, 29	16, 16	17, 22	14, 14	11, 15
T24 (ACC376)	XX	10, 12	12, 12	9, 9	10, 12	10, 11	6, 6	8, 11	17, 19	7, 10	16, 18	29, 29	16, 16	17, 22	14, 14	11, 15
T24* (ACC376)	XX	10, 12	9, 12	9, 9	10, 12	10, 11	6, 6	8, 11	17, 19							
T24* (HTB-4)	XX	10, 12	12, 12	9, 9	10, 12	10, 11	6, 6	8, 11	17, 17							

* Profile retrieved from DSMZ database (information is available for the first nine STR loci only).

In summary, UROtsa-1 and UROtsa-4 (and likely UROtsa-2) are authentic UROtsa cell line stocks, whereas UROtsa-3 and UROtsa/F35 are misidentified stocks that correspond to T24.

## Discussion

The UROtsa cell line is a valuable tool to study low-level effects of carcinogens and toxic substances in long-term experiments. For these studies it is important that the cell line itself does not undergo major changes during culturing. It is known that prolonged passaging can have a major impact on gene expression and other properties of a cell line [27]. This has to be taken into account when conducting experiments that require long-term culturing of cells. In addition, for cell lines that are not maintained by a central repository and that have been frequently exchanged between laboratories (like UROtsa) cumulated passage numbers are not always known.

UROtsa cells have been described to have a stable karyotype and showed no signs of malignant transformation in later passages [3]. Also, DNA methylation as well as cytokine and mRNA expression appeared to be mostly stable during culturing of normal UROtsa cells [11], [12], [21], [28]–[30]. In contrast, the expression of miRNAs during long-term culturing has not been published before.

Our analysis of a long-term experiment with a supposed UROtsa cell line stock (UROtsa-3) revealed moderate changes in the expression of several mRNAs as well as relatively small variations in the degree of DNA methylation. However, the chromosome numbers varied markedly and the RNA expression and methylation pattern were not typical for the molecular properties expected for UROtsa cells. At last, STR profiling proved that UROtsa-3 was actually a T24 cell line.

Initially, we noticed that for some genes the UROtsa-3 cells showed an unusual expression level, similar to an already transformed cell line. For example, authentic UROtsa cells are of transitional epithelial origin and should thus express markers like E-cadherin (*CDH1*), keratin 17 (*KRT17*), and tumor protein p63 (*TP63*) [31]–[34]. On the other hand, the mesenchymal marker zinc finger E-box binding homeobox 1 (*ZEB1*) was detectable and vimentin (*VIM*) showed a high expression, while members of the miR-200 family showed a relatively low expression. This was a first indication of a possible cross-contamination or at least a major change in the UROtsa-3 stock. To have a more suitable cells for comparison, we searched for UROtsa stocks that had not been infected or cross-contaminated before. In 2011, we were able to locate a UROtsa cell line stock (UROtsa-4) that apparently had never been contaminated and was kindly provided by A. Fabarius [5]. The mRNA and miRNA levels of the UROtsa-3 cells were clearly different from this authentic UROtsa-4 stock but similar to those of HeLa and T24 cells that served as controls. The relative mRNA expression levels of *ZEB1*, *CDH1*, *KRT17*, *VIM*, *TP63*, *TP53*, *RB1*, and *HRAS* in T24 have been published before and are basically in agreement with our results [32], [34]–[41]. The selected genes were initially chosen because they represent targets of SV40 large T-antigen (*TP53, RB1*), a proto-oncogene (*HRAS*), a marker for epithelial-to-mesenchymal transition (*ZEB1*), or markers for monitoring urothelial differentiation (*CDH1, KRT17, TP63, UPK1A*, etc.). Quantification of the miR-200 family was performed because in urothelial tissues *ZEB1* is regulated by miRNAs of this family [35], [42], [43]. Indeed, *ZEB1* and the miR-200 family were inversely correlated during the UROtsa-3 long-term experiment.

At that point it was not clear whether the UROtsa-3 cell line had changed over the years, e.g., during the Mycoplasma infection and/or its treatment, or had been cross-contaminated by another cell line.

Epigenetic changes like hypo- or hypermethylation of gene promoters and other genomic regions can be associated with early stages of tumorigenesis [44]. Jensen et al. found progressive changes in DNA methylation in UROtsa cells during long-term exposure to low levels of arsenicals [29]. One of our original goals was to confirm and expand these results. Instead, the atypical gene expression of UROtsa-3 prompted us to use the analysis of DNA methylation patterns to compare different cell lines in order to identify and characterize possible cross-contaminants. The methylation profiles based on eleven selected genes were unique for each cell line. UROtsa-3 shared some similarities with but was distinct from the T24 variant (ACC 376) we used as a control. It has to be noted, however, that UROtsa-3 was grown in a different medium than T24. The growth conditions can have a major impact on DNA methylation, gene expression, differentiation and other cellular properties [4], [45], [46]. Also, the UROtsa-3 stock had a different history and karyotype than the T24 control. The two separate samples of UROtsa-3 as well as UROtsa/F35 had a very similar methylation pattern, confirming their identical origin. Because sample B and UROtsa/F35 were first cultured three years later than sample A, a cross-contamination of all three samples in our lab appears unlikely.

The retroposon *LINE1* is normally methylated to about 60–70% in normal bladder and 50–55% in UROtsa, which is in agreement with our results for UROtsa-4 and HUEPC [47]. The other genes are mostly tumor suppressor genes that should have a low degree of promoter methylation (0–10%) in normal urothelium [48]–[54]. Methylation levels of 10–15% for *C1QTNF6* in UROtsa were reported before, being in good agreement with our value of 12% in UROtsa-4 [29].

The methylation results indicate that epigenetic changes might serve as an additional tool to distinguish cell lines and their variants [55]–[59]. Further research should be conducted to confirm this observation. To our knowledge, methylation profiling by pyrosequencing has not been used so far to characterize urothelial cell lines. However, Cabello et al. did a profiling of bladder cancer cell lines with MS-MLPA and Tellez et al. used pyrosequencing to profile melanoma cell lines [60], [61]. Methylation levels of *RARB, ERS1*, and *MGMT* in T24 were also determined by Cabello et al. and were similar to our results, despite the different methods used. Chan et al. reported the promoter of *RASSF1* to be fully methylated in T24, based on methylation-specific PCR, similar to the 93% methylation we found by pyrosequencing [62]. Aparicio et al., using pyrosequencing with T24 cells from ATCC, found a methylation level of 37% in *LINE1* compared to 47% in our T24 cell line [63].

Because changes in gene expression levels as well as DNA methylation can, in principle, be reversible, we next looked for more fundamental changes, i.e. on the genomic level.

Since the original UROtsa cells were immortalized with an SV40 large T-antigen construct, detection of the *SV40gp6* gene appeared to be an easy way to distinguish the cells from classical contaminants like T24 and HeLa. The lack of *SV40gp6* in authentic UROtsa stocks (UROtsa-1 and UROtsa-4), however, came as a surprise. Petzoldt et al. verified the expression of large T-antigen protein in their immortalized cells within the first 15 passages. However, they already mentioned in their original publication that despite having used a temperature-sensitive variant of *SV40gp6* the growth of the immortalized cells was not temperature-sensitive [3]. They speculated that the integration of the SV40 construct itself might have activated a gene necessary for continuous cell growth, rendering a functional *SV40gp6* unnecessary. The *SV40gp6* DNA may have been lost during culturing of the UROtsa cells in the following months or years. Because we did not detect any *SV40gp6* DNA in two independent strains of UROtsa that date back to 1999 and 2001, we assume that the loss of large T-antigen happened before 1999. A loss of *SV40gp6* sequences has been described before for a neuroectodermal tumor cell line [64].

Since its establishment in 1973, changes of the karyotype of the T24 cell line have been described, some resulting in different variants [38], [65]–[69]. The original T24 cell line was hypotetraploid, which changed to triploid in long-term culture. The derived sublines were cytogenetically more homogeneous [66]. ATCC reports 86 chromosomes for the stemline of their T24 cell line HTB-4 (www.lgcstandards-atcc.org), while DMSZ reports 70–77 chromosomes for their ACC 376 cell line (www.dsmz.de). The number of chromosomes of the UROtsa-3/T24 cell line varied between 55 and 83, with a median of 80 chromosomes in an early passage. Whether this represents a new variant of T24 remains to be determined.

T24 cells are a frequent contaminant that is only ‘outperformed’ by HeLa cells [17]. The following cell lines, among others, are known to be identical to or variants of T24: ACCS, EJ (EJ1, MGH-U1), EJ138, ECV304, GHE, HAG, HU456, HU961T, JCA-1, MGH-U2, TSU-Pr1, and UM-UC-2 (see also www.cellbankaustralia.com) [16], [17], [20], [70]–[72]. In addition, some stocks of J82 have been contaminated by T24 in the past. T24 is a popular cell line used in many labs. When working with urothelial (cancer) cell lines, T24 is frequently used for comparison und should therefore always be considered as a source of contamination.

The use of misidentified or cross-contaminated cell lines is still widespread. It is estimated that 15–36% of cell lines are incorrectly designated [13], [20], [72]–[74]. A survey by Buehring et al. in 2004 revealed that 9.5% of cell lines were HeLa contaminants. About 46% of the surveyed laboratories did not use any appropriate methods for cell line identification and 35% obtained cell lines from other laboratories rather than cell banks, which are more likely to have quality control programs in place [75].

It is difficult to determine the time and place where the T24 contamination of UROtsa occurred. The original UROtsa cells went to West Virginia University and NIOSH from where they were given to UNC in 2000 [76], [77]. Because the derived cell line UROtsa/F35 was created in 2004, we assume that the cross-contamination occurred before 2004 (Z. Drobna, personal communication and [25]). In cases where the contaminated UROtsa cells have been used only as a cellular model for gene delivery or gene knockdown, the mix-up should not make a difference. However, in studies that used UROtsa as a (non-cancer) control the results should be reevaluated. We have notified all laboratories that – to our knowledge – have received samples of the cross-contaminated UROtsa cells. Nevertheless, it is possible that the UROtsa-3/T24 cell line stock is still in use in some laboratories. It is therefore important to check existing stocks of UROtsa, especially when embarking on major studies like the ENCODE project [58].

Currently, the UROtsa cell line is not available from cell banks. Based on our results of the tested stocks we recommend UROtsa-4 as an authentic and Mycoplasma-free UROtsa cell line stock (available, e.g., in the laboratories of A. Fabarius, Mannheim and E. Dopp, Essen, Germany). However, that does not exclude the availability of suitable stocks from other laboratories [78].

To avoid the use of false cell lines, we would recommend the following steps:

1. Before working with a new cell line, databases (e.g., www.cellbankaustralia.com) and current publications (e.g., www.ncbi.nlm.nih.gov/pubmed) should be checked for possible cross-contaminated or misidentified cell lines [17].
2. STR profiling of each new stock should be performed before starting any experiment. If performing long-term culturing, additional checks during and after an experiment are recommended. Results should be compared to a database of cell line STR profiles (e.g., www.dsmz.de or www.atcc.org) [18].
3. In general, deposition of new cell lines in cell banks would greatly reduce the number of misidentified cell lines because cell banks have all necessary tools to characterize cells and test for authenticity [13]. They also ensure, through regular checks, the quality of their distributed cell lines. In cases where laboratories choose not to deposit a new cell line, the STR profile can still be contributed, for example, to the BioSample database (www.ncbi.nlm.nih.gov/biosample) [74].

An investment in these preventive steps would be small compared to the waste of money, time, and resources associated with research on false cell lines [75].

Several lines of evidence – based on RNA expression, DNA methylation, and genomic analyses – proved that the UROtsa-3 cell line stock is identical to T24. Cross-contamination of cell lines remains a serious problem. Especially cell lines that are not well documented and have not been submitted to cell banks pose a higher risk of a contamination that might remain hidden for years. Regular identity checks of cell lines, e.g., before, during, and after long-term experiments, are highly recommended. The presented results should simplify the verification of UROtsa cell lines and help to avoid cross-contaminations in the future. Aberrations on different molecular levels may serve as indicators of a possible contamination. DNA methylation profiling appears to be a promising tool for a more detailed molecular characterization of cell lines. An interesting finding is that authentic UROtsa cells appear not to harbor any large T-antigen. In future studies, the interpretation of the molecular behavior of UROtsa cells should therefore consider this fact. The contaminating T24 cell line had varying chromosome numbers, but showed only relatively moderate changes in the expression and methylation of selected genes. This has to be taken into account when using this cell line stock for long-term experiments.

## Supporting Information

Figure S1 **Time course of mRNA expression in UROtsa-3/T24 cells.** Shown are the normalized mRNA levels of *VIM* (green), *HRAS* (pink), *RB1* (blue), *ZEB1* (dark blue), *TP53* (light blue), *NOTCH1* (grey), and *KRT17* (orange) as determined by Real-Time PCR. *GAPDH* was used for normalization. *CDH1*, *KRT20* and *TP63* were not detectable. For *UPK1A* only a few time points were detectable (trace not shown).
(TIF)

Figure S2 **Time course of miRNA expression (miR-200 family) in UROtsa-3/T24 cells.** The normalized levels of miR-200a (red), miR-200b (green), miR-200c (blue), and miR-429 (orange) are shown as determined by Real-Time PCR. RNU44 and RNU48 levels were used for normalization. MiR-141 was not detectable.
(TIF)

Figure S3 **Time course of DNA methylation of several genes in UROtsa-3/T24 cells.** The degree of promoter methylation of the genes *PGR, RASSF1*, *RARB, LINE1, FHIT, ESR1, CDH1, C1QTNF6, MGMT, PTGS2*, and *SOCS3* was determined by pyrosequencing. Samples were taken every four weeks during long-term culturing of UROtsa-3 cells.
(TIF)

Figure S4 **STR profile analysis of the cell line stocks UROtsa-1, UROtsa-4, UROtsa-3/T24, and the cell line T24.**
(PDF)

Table S1 **Real-Time PCR results of the mRNA expression analysis of eleven different genes and the expression analysis of members of the miR-200 family.**
(XLSX)

Table S2 **Details of PCR conditions and primer sequences for the DNA methylation analysis.**
(XLSX)
