# Supplementary figures and images for: Cross-Contamination of a UROtsa Stock with T24 Cells – Molecular Comparison of Different Cell Lines and Stocks
Source: PLoS One. 2013 May 17;8(5):e64139. doi: 10.1371/journal.pone.0064139 (PMC3656924; doi:10.1371/journal.pone.0064139)

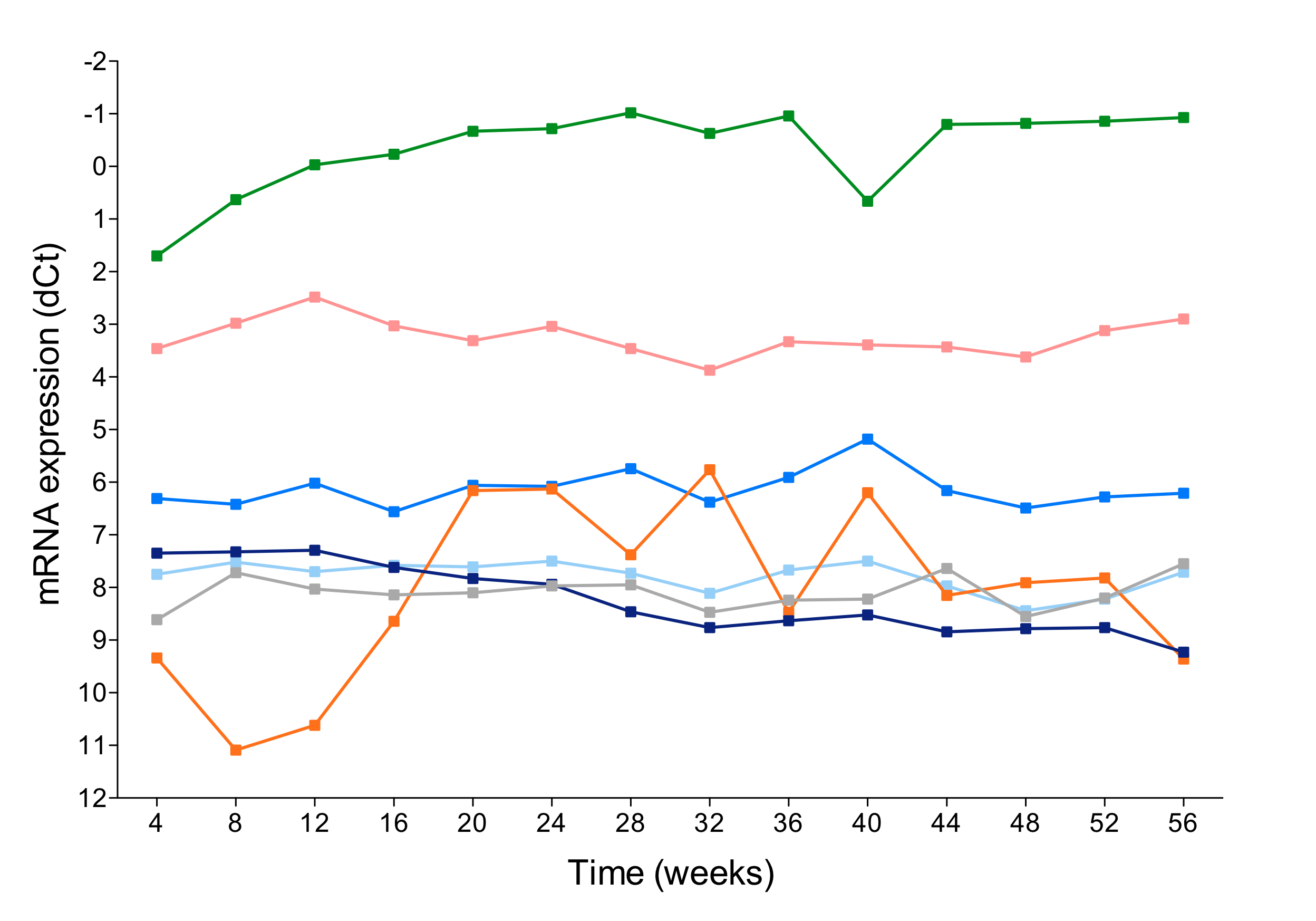

Supplement: Figure S1 — Time course of mRNA expression in UROtsa-3/T24 cells. Shown are the normalized mRNA levels of VIM (green), HRAS (pink), RB1 (blue), ZEB1 (dark blue), TP53 (light blue), NOTCH1 (grey), and KRT17 (orange) as determined by Real-Time PCR. GAPDH was used for normalization. CDH1, KRT20 and TP63 were not detectable. For UPK1A only a few time points were detectable (trace not shown). (TIF) [file pone.0064139.s001.tif]

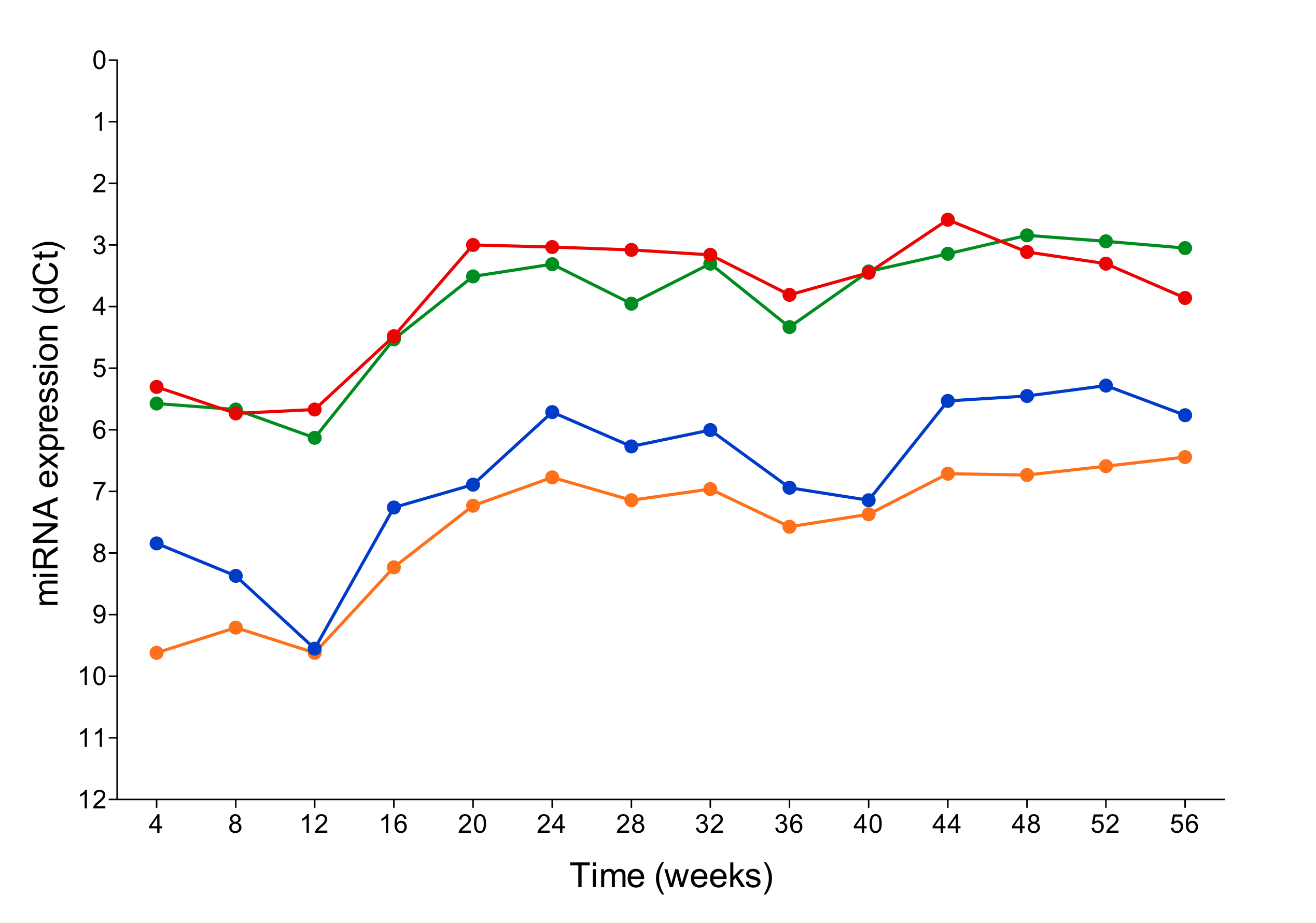

Supplement: Figure S2 — Time course of miRNA expression (miR-200 family) in UROtsa-3/T24 cells. The normalized levels of miR-200a (red), miR-200b (green), miR-200c (blue), and miR-429 (orange) are shown as determined by Real-Time PCR. RNU44 and RNU48 levels were used for normalization. MiR-141 was not detectable. (TIF) [file pone.0064139.s002.tif]

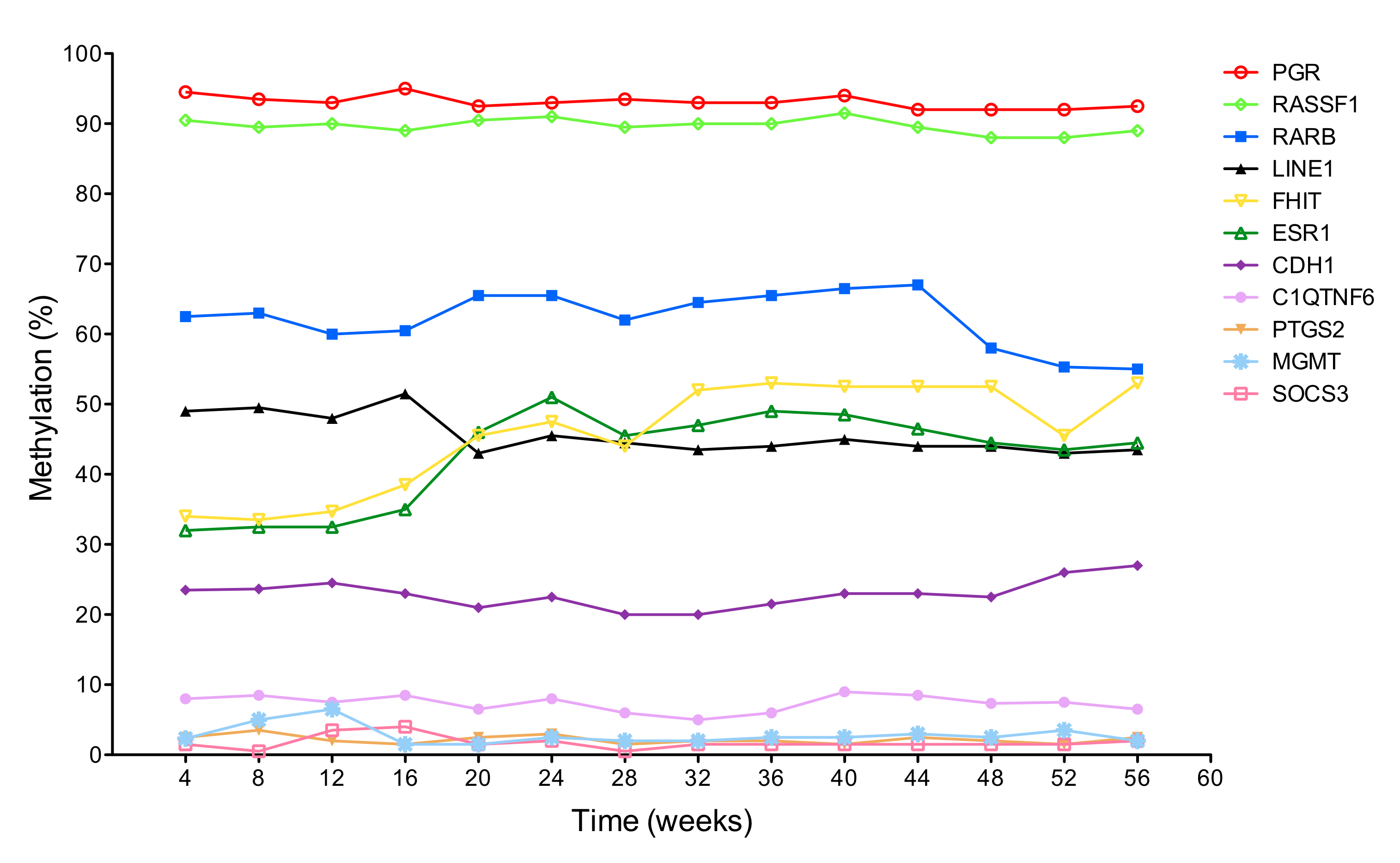

Supplement: Figure S3 — Time course of DNA methylation of several genes in UROtsa-3/T24 cells. The degree of promoter methylation of the genes PGR, RASSF1, RARB, LINE1, FHIT, ESR1, CDH1, C1QTNF6, MGMT, PTGS2, and SOCS3 was determined by pyrosequencing. Samples were taken every four weeks during long-term culturing of UROtsa-3 cells. (TIF) [file pone.0064139.s003.tif]

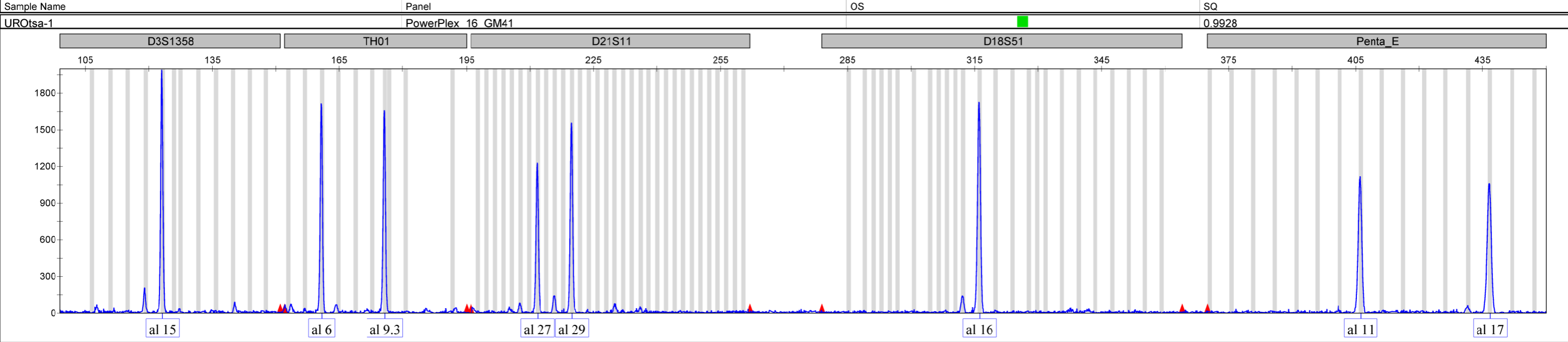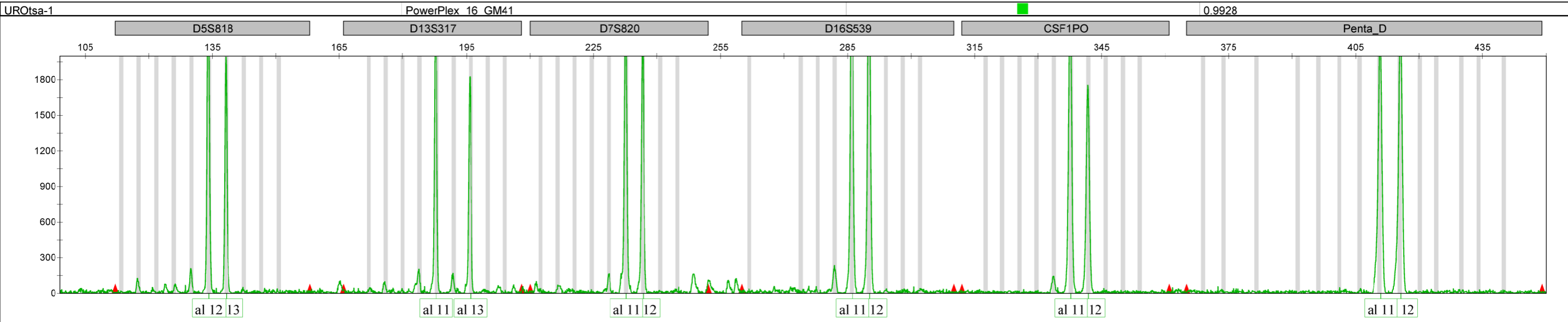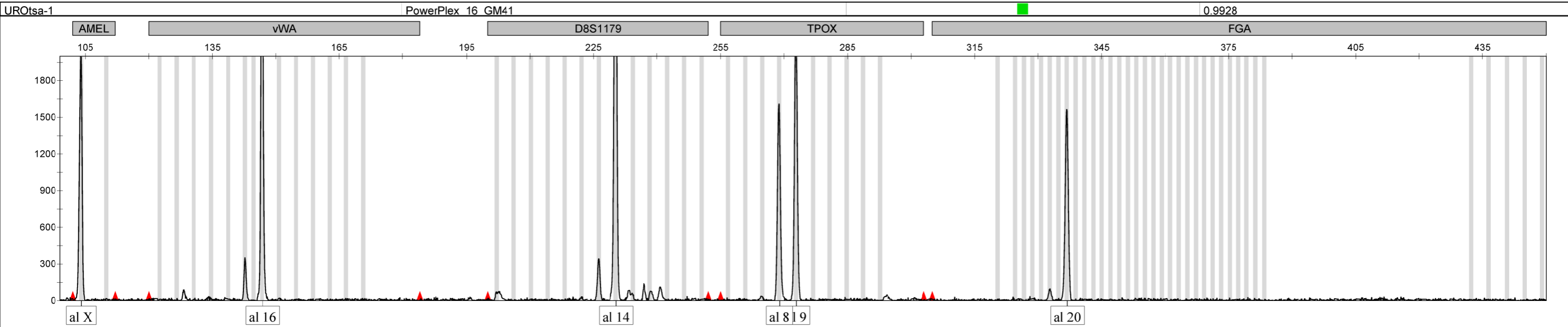

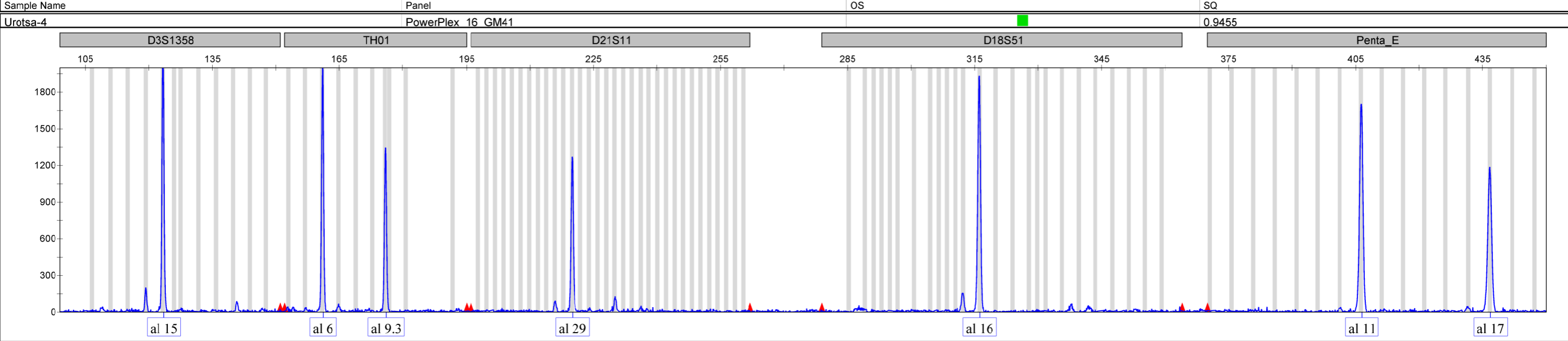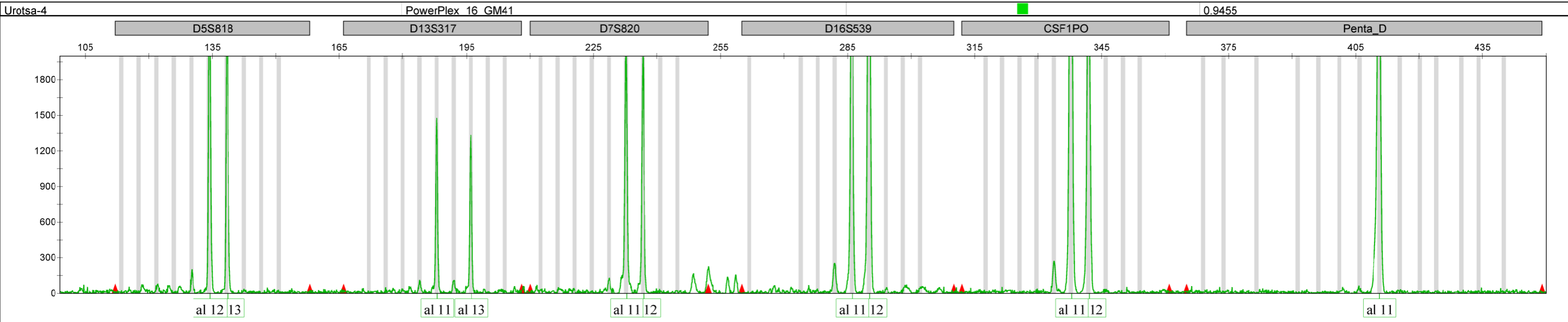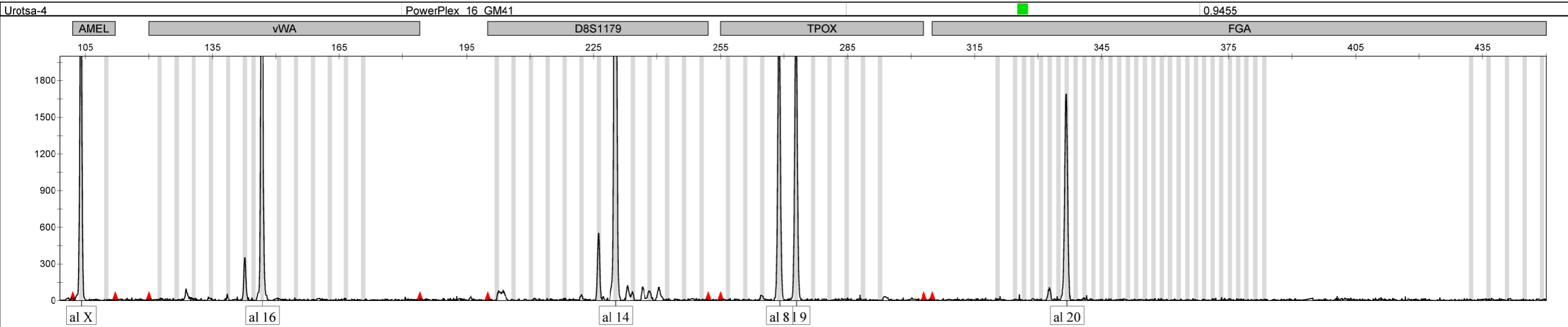

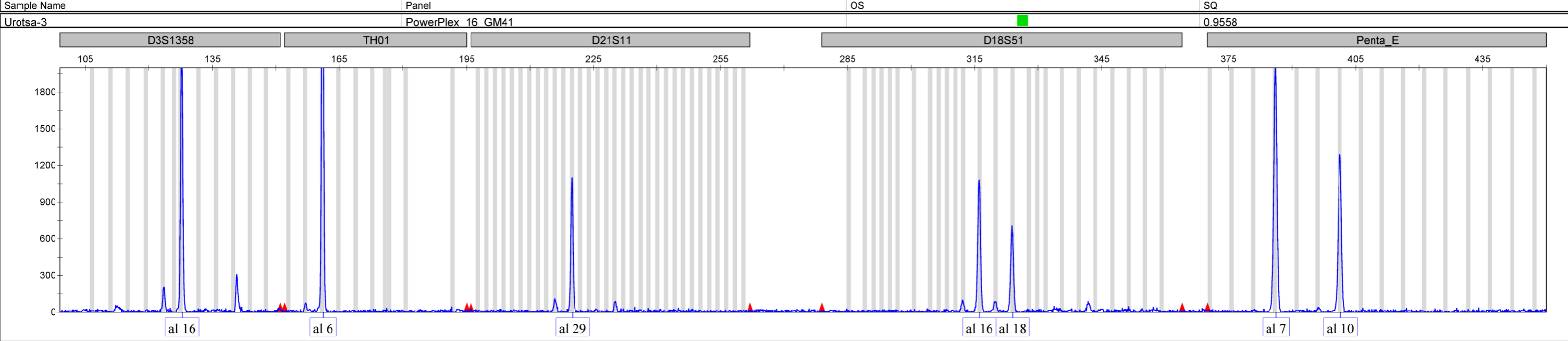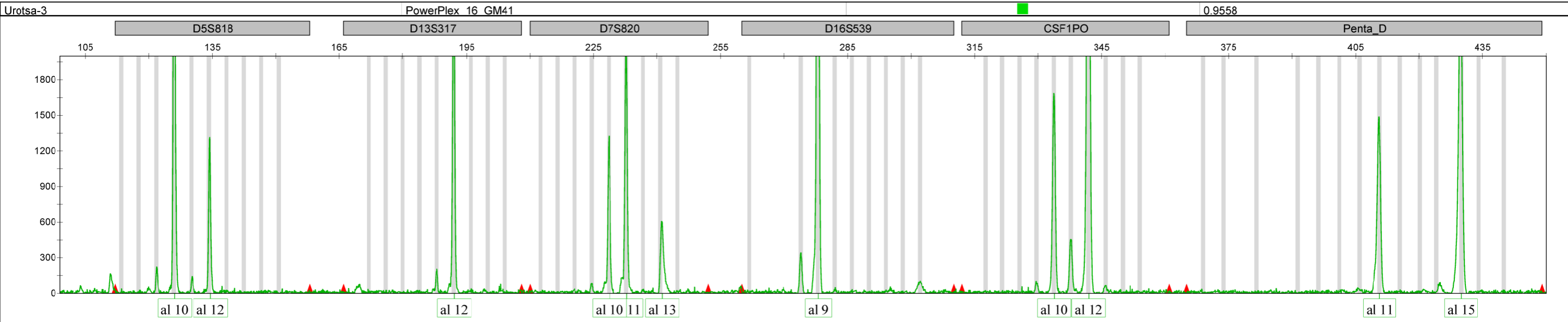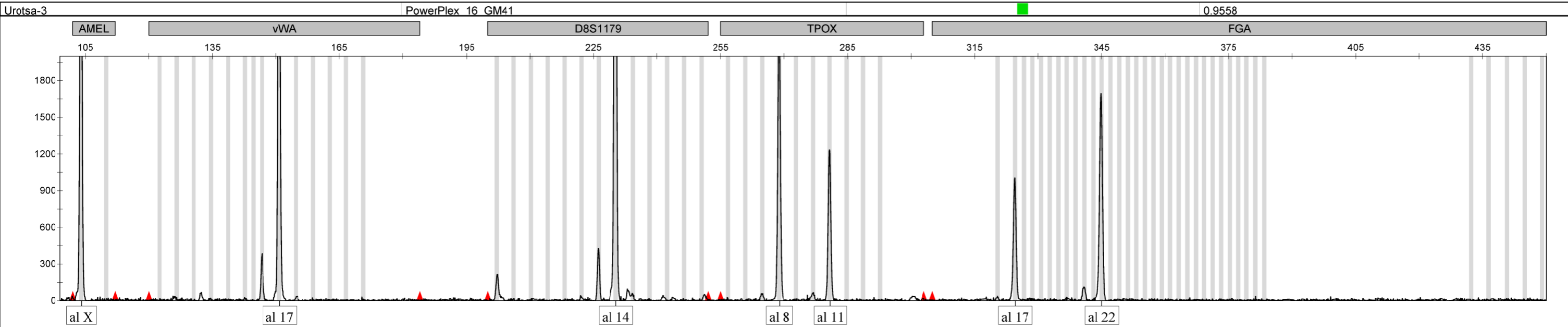

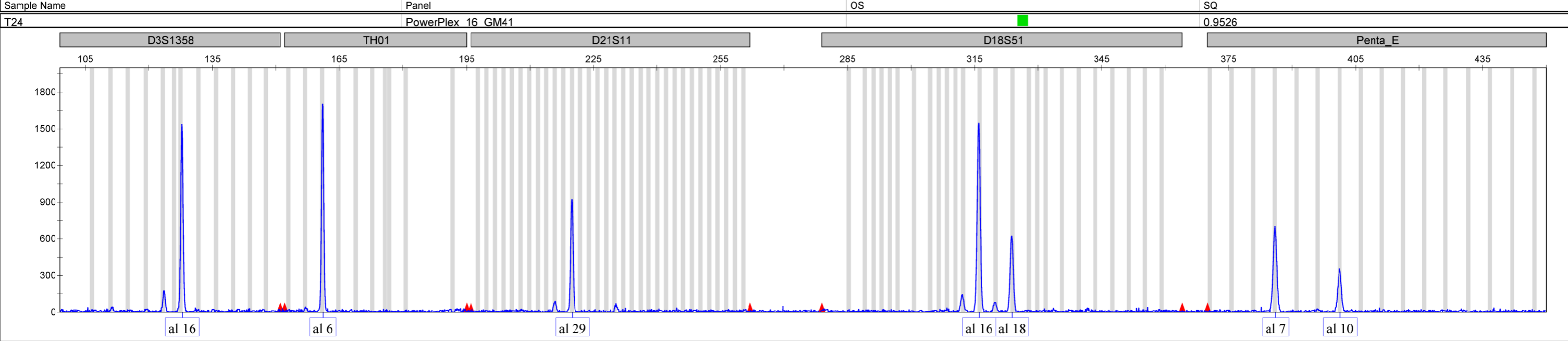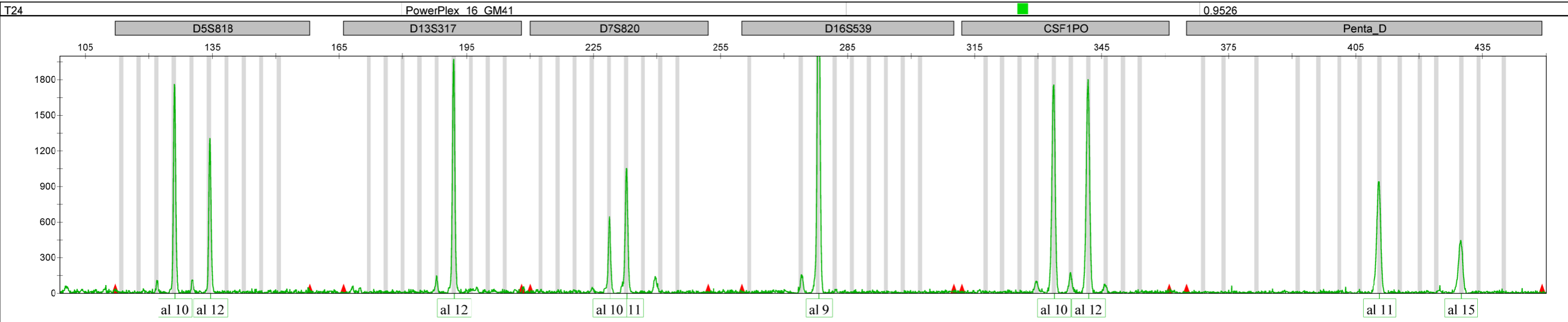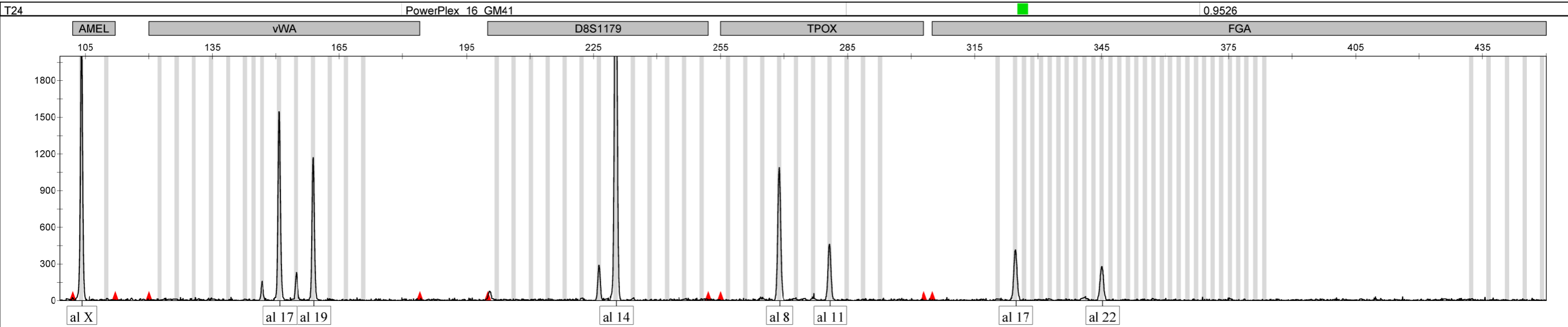

Supplement: Figure S4 — STR profile analysis of the cell line stocks UROtsa-1, UROtsa-4, UROtsa-3/T24, and the cell line T24. (PDF) [file pone.0064139.s004.pdf › Johnen_T24-UROtsa STR FigS4.pdf]
